# Technical factors associated with biliary cannulation success after transpancreatic precut sphincterotomy

**DOI:** 10.1055/a-2863-1217

**Published:** 2026-05-19

**Authors:** Wei-Chih Su, Tsung-Hsien Hsiao, Chia-Chi Wang, Hung-Da Chen, Tzu-Hsiang Kung, Chih-Hsiang Chen, Jiann-Hwa Chen

**Affiliations:** 1 Department of Gastroenterology 145204 Taipei Tzu Chi HospitalTaipei CityTaiwan; 2 School of Medicine 59216 Tzu Chi UniversityHualienTaiwan

**Keywords:** Pancreatobiliary (ERCP/PTCD), ERC topics, Quality and logistical aspects, Training, Performance and complications

## Abstract

**Background and study aims:**

Transpancreatic precut sphincterotomy (TPS) is a rescue technique for difficult biliary cannulation, but the factors associated with its success remain incompletely defined. In this study, patient-related and technical variables associated with biliary access after TPS were evaluated.

**Methods:**

We retrospectively reviewed 92 endoscopic retrograde pancreatography (ERCP) procedures requiring TPS between 2016 and 2023. Because additional incisions could be created after unsuccessful attempts, a total of 115 TPS sessions were analyzed. Patient characteristics and technical parameters, including papilla morphology, incision extent, and exposure, were assessed. Incision extent was categorized using the midpoint of the papillary oral protrusion as a visual landmark and the post-TPS orientation of bile and pancreatic orifices was documented.

**Results:**

The overall biliary cannulation success rate was 93.5%. In the session-level analyses, Haraldsson type 2 papillae were associated with lower success, whereas long incisions, defined as those extending beyond the midpoint, were independently associated with higher success (odds ratio 3.71, *P* = 0.010). At the ERCP level, initiating TPS with a long/full incision was associated with a higher first incision cannulation rate and shorter cannulation time without an increase in adverse events. After the TPS, the bile duct orifice was most often located in the upper-left region of the pancreatic orifice with a distance less than two sphincterotome widths (65.9%).

**Conclusions:**

TPS outcomes are associated mainly with technical factors, with type 2 papillae showing reduced success. Longer incisions were associated with higher biliary access rates and the bile duct orifice typically was upper-left of the pancreatic orifice after TPS.

## Introduction

Endoscopic retrograde cholangiopancreatography (ERCP) requires a high rate of selective biliary cannulation, yet standard cannulation techniques achieve success in only approximately 77.3% to 83.6% of procedures 1 , which remains below the 90% benchmark recommended by the European Society of Gastrointestinal Endoscopy 2 . As a result, rescue cannulation techniques are needed in a substantial proportion of ERCPs.

Transpancreatic precut sphincterotomy (TPS) is one of the three ESGE-endorsed approaches for difficult biliary access 3 . TPS involves guidewire-assisted pancreatic duct cannulation followed by incision of the septum between the pancreatic and bile ducts to facilitate biliary entry. Compared with the needle-knife precut, the TPS is generally regarded as a more controlled and reproducible technique 4 . Since its first description by Goff in 1995 5 , accumulating evidence has supported its efficacy. A meta-analysis reported a 91.7% cannulation rate and a 7.1% incidence of post-ERCP pancreatitis (PEP) 6 and a recent network meta-analysis demonstrated that TPS had both the highest cannulation success and the lowest PEP risk among modern rescue techniques 7 .

Despite these data, compared with other advanced cannulation techniques, TPS remains less thoroughly evaluated, particularly with respect to technical factors that determine its success. Existing reports have not clearly defined how incision extent, incision exposure, or patient-related characteristics influence outcomes, and most have focused only on overall cannulation rates rather than the stepwise performance of TPS. Clarifying how these technical elements are related to biliary access may help reduce operator-dependent variability and improve procedure consistency. At our center, which performs approximately 200 therapeutic ERCPs annually, TPS has been the primary rescue technique for difficult biliary cannulation since 2016. This study, therefore, examined both technical parameters and patient-related factors associated with TPS success using a combined session-level and procedure-level analytic approach to provide a more detailed assessment of determinants of biliary access.

The study was approved by the Institutional Review Board (12-X-042) of Taipei Tzu Chi Hospital, Buddhist Tzu Chi Medical Foundation.

## Methods

**Fig. 1:**
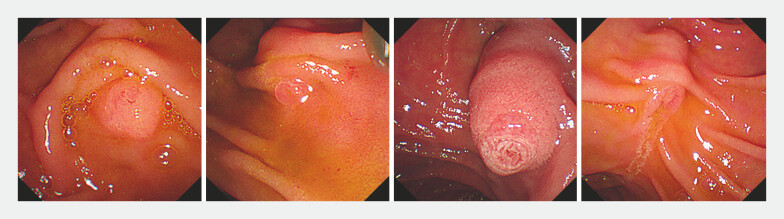
From left to right: Type 1. Regular papilla, with no distinctive features; Type 2. Small papilla, often flat, with a diameter not bigger than 3 mm (approximately 9F); Type 3. Protruding or pendulous papilla. A papilla is standing out, protruding or bulging into the duodenal lumen or sometimes hanging down, pendulous, with the orifice oriented caudally; Type 4. Creased or ridged papilla, where the ductal mucosa seems to extend distally out of the orifice either on a ridge or in a crease.

We retrospectively reviewed ERCP procedures requiring TPS from April 2016 to March 2023. The collected data included demographics, clinical characteristics, ERCP findings, and procedure outcomes. ERCP indications were categorized as biliary stones, distal obstruction, proximal obstruction, or other causes. Papilla morphology was classified according to the Haraldsson system: type 1, regular; type 2, small with an orifice ≤ 3 mm; type 3, enlarged or protruding; and type 4, creased or ridged 8 ( Fig. 1 ). Periampullary diverticula were classified using the method of Shi et al. 9 . All classifications were based on stored duodenoscopic images.

### ERCP procedure

All ERCPs were initiated with standard cannulation under monitored anesthesia. Six experienced endoscopists performed the procedures: one high-volume operator (approximately 80 therapeutic ERCPs per year) and five low-volume operators (20 to 30 per year). Difficult cannulation was defined as cannulation time exceeding 5 minutes. TPS was the preferred rescue technique when deep pancreatic duct access was achieved, whereas needle-knife precut was used when pancreatic access was not possible or when the papilla was caudally oriented. The double-guidewire technique was rarely used. TPS was performed when selective bile duct cannulation failed after more than 5 minutes and when pancreatic duct access was achievable. Wire-guided sphincterotomy was performed at the 11 o’clock position using a sphincterotome (CleverCut, Olympus Medical Systems, Tokyo, Japan; or TRUEtome, Boston Scientific, Marlborough, Massachusetts, United States). A pancreatic stent was usually placed, although omission occurred at operator discretion. Bile duct cannulation was attempted along the left cutting edge of the incision, with additional incisions performed when necessary. TPS was not standardized at our institution and incision extent was determined by the operator. Prophylaxis for PEP, including hydration with 1000 mL of lactated Ringer’s infused over 8 hours starting 15 minutes before ERCP and rectal nonsteroidal anti-inflammatory drugs (NSAIDs), was implemented in November 2018.

### Definitions and data measurement

**Fig. 2:**
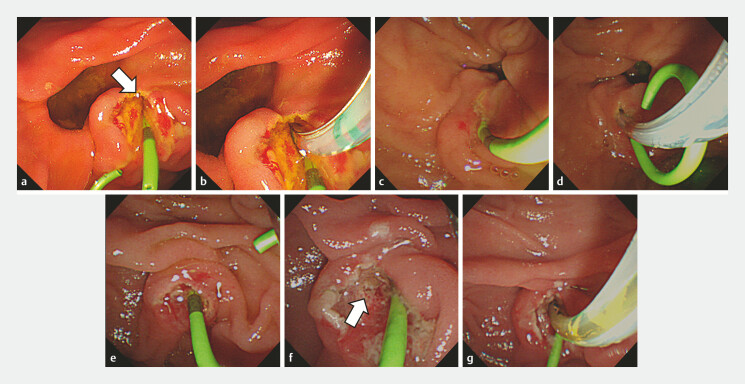
Difficult bile duct cannulation cases managed with transpancreatic precut sphincterotomy (TPS). **a** TPS was performed with a full incision extending to the superior margin of the papillary oral protrusion; the incision site was well-exposed. The bile duct orifice is visible at the upper left of the incision (white arrow). **b** Successful selective bile duct cannulation was achieved on the left side of the incision. c Long incision extending beyond the midpoint of the papillary oral protrusion with inadequate exposure. **d** Successful cannulation was achieved after the guidewire was probed on the upper-left side of the incision. **e** A short initial incision proximal to the midpoint of the papilla where bile duct cannulation failed after the TPS. f In a subsequent session, extension of the incision revealed the bile duct orifice at the left margin (white arrow). **g** Successful bile duct cannulation using a sphincterotome.

Additional TPS incisions were performed when the initial incision did not achieve biliary access, resulting in more than one TPS session in some procedures. A TPS session was defined as all cannulation attempts following a single incision. When multiple incisions occurred during the same ERCP ( Fig. 2 **e-g** ), only the first and final incisions were analyzed; intermediate incisions were excluded because documentation was incomplete and reproducibility could not be ensured. Analyses were conducted at two levels: the session level, evaluating factors associated with success of each TPS session, and the ERCP level, assessing overall procedure performance and outcomes.

**Fig. 3:**
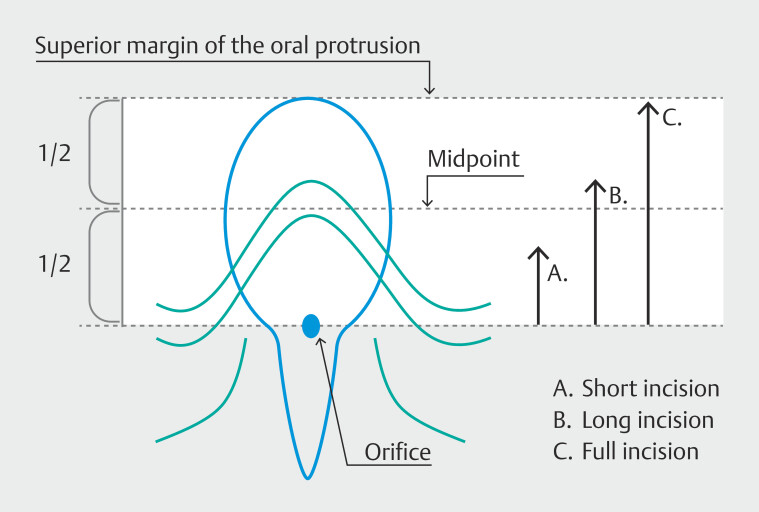
The midpoint of the papillary oral protrusion was used as a reproducible endoscopic landmark to define the extent of the TPS incision: A. short, ending below the midpoint; B. long, reaching or exceeding the midpoint; and C. full, reaching the superior margin. Dashed lines indicate the reference levels.

**Fig. 4:**
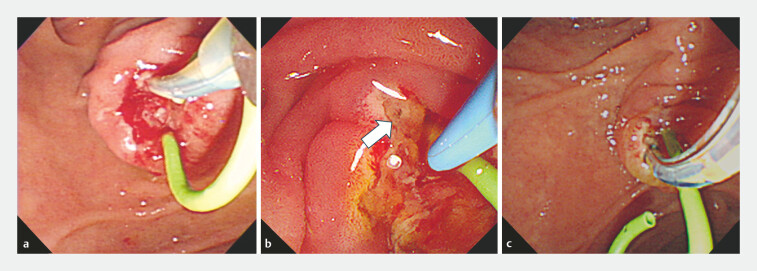
Relative positions of the bile duct and pancreatic duct following successful cannulation after transpancreatic precut sphincterotomy were classified into three types: **a** upper left with a distance exceeding twice the width of the sphincterotome, **b** upper left with a distance less than twice the width of the sphincterotome, and c left and lower left.

Incision extent was retrospectively classified using stored endoscopic images obtained before and after sphincterotomy. Images captured at initial papilla visualization were first reviewed to identify the papillary orifice and the visually identifiable superior margin of the oral protrusion. On the basis of these endoscopic landmarks, the midpoint between the orifice and the superior margin was visually estimated. Extent of the TPS incision was then determined using post-incision images and categorized according to its relation to this midpoint reference ( Fig. 3 ): short, ending proximal to the midpoint; long, reaching or exceeding the midpoint; or full, reaching the superior margin. Incision exposure, defined as visibility of the cutting edge, was classified as well-exposed ( Fig. 2 **a** , **b** ) or inadequately exposed ( Fig. 2 **c** , **d** ). In successfully cannulated cases, the position of the bile duct orifice relative to the pancreatic duct orifice was documented and categorized as follows: type A, upper-left with a distance greater than two sphincterotome widths; type B, upper-left with a distance less than two widths; and type C, left or lower left ( Fig. 4 ).

Acute bleeding after TPS or any sphincterotomy that obscured the endoscopic view was recorded separately and persistent bleeding requiring hemostasis was also documented. Recorded variables included common bile duct cannulation time, total ERCP duration, and ERCP-related adverse events (AEs) (PEP, cholangitis, delayed bleeding, and perforation). PEP was defined as abdominal pain with a lipase level at least three times the upper limit of normal more than 24 hours after the procedure, accompanied by a prolonged hospital stay 10 . Cholangitis was defined as fever greater than 38°C with a biliary source and without acute cholecystitis. Delayed bleeding was defined as gastrointestinal bleeding (melena, hematemesis, or hemoglobin drop ≥ 2 g/dL) or need for transfusion. Perforation was limited to sphincterotomy-related injuries identified by extraluminal air on imaging. Severity of ERCP-related AEs was graded according to the consensus criteria proposed by Cotton et al. 10 .

Endoscopic images were captured at papilla arrival, after each sphincterotomy, and at successful cannulation. Fluoroscopic images were obtained during deep guidewire insertion and at the end of the examination. CBD cannulation time and total ERCP duration were measured from first visualization of the papilla to successful cannulation and to the end of the procedure, respectively. Two experienced endoscopists independently reviewed the stored duodenoscopic images. Papilla type and diverticulum classification were determined on the basis of images captured at initial papilla visualization. Reviewers were blinded to procedure outcomes, including cannulation success and AEs, at time of assessment. Incision extent and exposure status were evaluated retrospectively using images obtained after sphincterotomy. Because these images reflected the procedure course, completely blinding to procedure outcomes was not entirely feasible. However, the reviewers did not have access to procedure records or outcome data and classification was performed according to predefined endoscopic landmarks. Discrepancies between the reviewers were resolved by consensus with a third endoscopist.

### Statistical analysis

Categorical variables are presented as numbers with percentages and continuous variables as means with standard deviations. The chi-square test or Fisher’s exact test was used for categorical variables and Student’s *t* test for continuous variables. Logistic regression was used to evaluate factors associated with successful bile duct cannulation. Multivariable analysis was performed using a backward stepwise logistic regression approach to identify factors that are independently associated with TPS session success. Clinically relevant demographic, anatomical, and procedural variables, including age, sex, indication, papilla morphology, periampullary diverticulum subtype, endoscopist volume, incision extent, incision exposure, and acute post-incision bleeding, were initially entered into the model. Given the limited number of failure events, model complexity was intentionally constrained to minimize overfitting in accordance with the events-per-variable principle. Sensitivity analyses were performed by forcibly entering examiner volume and calendar time into the multivariable logistic regression model to assess potential operator-related confounding and learning curve effects. Calendar time was treated as an ordinal variable representing sequential annual intervals from introduction of TPS at our institution (coded 1–7). Interobserver agreement for papilla type, incision extent, and incision exposure was assessed using Cohen’s kappa, with weighted kappa applied to the ordinal variable of incision extent. Agreement strength followed the criteria of Landis and Koch. All analyses were performed using SPSS version 27.0 (IBM Corp., Armonk, New York, United States). Two-tailed *P* < 0.05 was considered statistically significant.

## Results

**Fig. 5:**
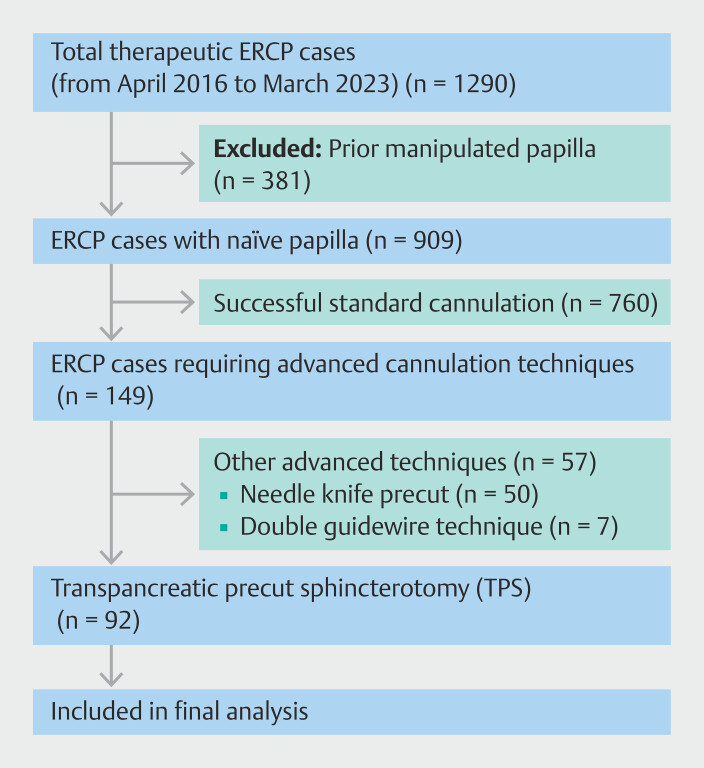
Study flowchart of ERCP case selection during the study period.

From April 2016 to March 2023, 1,290 therapeutic ERCP procedures for biliary diseases were performed at our institution, including 909 involving naïve papillae. Among these, 149 required advanced cannulation techniques, including TPS in 92 patients, needle-knife precut in 50 patients, and the double-guidewire technique in seven patients ( Fig. 5 ). The 92 patients who underwent TPS were included in the present analysis. Interobserver agreement was moderate for papilla type (κ = 0.43; 95% confidence interval [CI] 0.24–0.62), substantial for TPS incision extent (weighted κ = 0.77; 95% CI 0.64–0.90), and almost perfect for incision exposure (κ = 0.85; 95% CI 0.74–0.96).

As shown in Table 1 , 47 patients (51.1%) were male and 75 (81.5%) were older than 50 years. The most common indication was biliary stones (75.0%), followed by distal (13.0%) and proximal (9.8%) biliary obstruction; two patients (2.2%) underwent ERCP for bile leakage after cholecystectomy. Fourteen patients (15.2%) had biliary pancreatitis and three (3.3%) had end-stage renal disease. Among patients who underwent TPS, Haraldsson type 1 papillae were most common (30.4%), followed by type 3 (29.3%), type 4 (25.0%), and type 2 (15.2%). A periampullary diverticulum was present in approximately one-third of patients and a high-volume endoscopist performed half the procedures. Pancreatic stents were placed in 84 patients (91.3%), not placed in six, and migrated in two. The overall bile duct cannulation success rate was 93.5%. The initial TPS incision achieved biliary access in 67 patients (72.8%), whereas 23 (25.0%) required additional incisions. PEP developed in seven cases (7.6%). Acute post-TPS bleeding occurred in nine patients (9.8%), 19 patients (20.7%) experienced bleeding after any sphincterotomy, and eight (8.7%) required endoscopic hemostasis. Delayed bleeding occurred in five patients (5.4%). Cholangitis was observed in four patients (4.3%). All cases of PEP, bleeding, and cholangitis were graded as mild. One sphincterotomy-related perforation was graded as moderate and was successfully managed conservatively.

**Table:** **Table 1** Patient characteristics and outcomes of transpancreatic precut sphincterotomy.

**Number of patients**	**92**
**Male**	47 (51.1%)
**Age > 50**	75 (81.5%)
**Indication**	
Biliary stone	69 (75.0%)
Distal obstruction	12 (13.0%)
Proximal obstruction	9 (9.8%)
Others	2 (2.2%)
**History of biliary pancreatitis**	14 (15.2%)
**End-stage renal disease**	3 (3.3%)
Macroscopic appearance of the ampulla Vater*	
Type 1	28 (30.4%)
Type 2	14 (15.2%)
Type 3	27 (29.3%)
Type 4	23 (25.0%)
**Periampullary diverticulum†**	
No diverticulum	62 (67.4%)
Type I	1 (1.1%)
Type IIa	7 (7.6%)
Type IIb	12 (13.0%)
Type III	10 (10.9%)
**Endoscopist**	
High volume	46 (50.0%)
Low volume	46 (50.0%)
**Post-ERCP pancreatitis prevention**	
Pancreatic stenting	84 (91.3%)
Peri-ERCP hydration	59 (64.1%)
Rectal NSAIDs ^‡^	38 (41.3%)
**Successful biliary cannulation**	86 (93.5%)
Successful cannulation after initial TPS ^§^ incision	67 (72.8%)
**Requirement of additional incision**	23 (25.0%)
**ERCP outcomes**	
Cannulation time	28.5 (14.2) ^¶^
ERCP procedure time	49.1 (19.3) ^¶^
Post-ERCP pancreatitis	7 (7.6%)
Active post-pancreatic sphincterotomy bleeding	9 (9.8%)
Post-sphincterotomy bleeding, any cause	19 (20.7%)
Bleeding requiring hemostasis	8 (8.7%)
Delayed bleeding	5 (5.4%)
Cholangitis	4 (4.3%)
Perforation	1 (1.1%)
*Type 1: regular papilla; Type 2: small papilla, often flat, with a diameter ≤ 3 mm; Type 3: protruding or pendulous papilla; Type 4: creased or ridged papilla. ^†^ Papilla located completely inside the diverticulum (type I), papilla located in the inner (type IIa) and outer (type IIb) margins of the diverticulum; and papilla located outside the diverticulum (type III). ^‡^ Nonsteroidal anti-inflammatory drugs. ^§^ Transpancreatic precut sphincterotomy. ^¶^ Mean (standard deviation). ERCP, endoscopic retrograde cholangiopancreatography; NSAID, nonsteroidal anti-inflammatory drug	

A total of 115 TPS sessions were recorded. Table 2 summarizes patient characteristics, technical factors, and cannulation outcomes. Fifty-three sessions (46.1%) were performed in male patients and 96 (83.5%) in patients older than 50 years. Distribution of papilla types was 29.6% for type 1, 16.5% for type 2, 27.8% for type 3, and 26.1% for type 4. Periampullary diverticulum was absent in 77 sessions (67.0%) and a high-volume endoscopist performed 57 sessions (49.6%). Incision exposure was well-exposed in 62 sessions (53.9%). Incision extent was short in 52 sessions (45.2%), long in 53 (46.1%), and full in 10 (8.7%). The overall biliary cannulation success rate per session was 74.8%. Success was higher with well-exposed than inadequately exposed incisions (82.3 vs. 66.0%). Cannulation rates were 59.6% for short incisions, 86.8% for long incisions, and 90.0% for full incisions. Long incisions achieved significantly higher success rates than short incisions did ( *P* = 0.003), whereas long and full incisions had similar success rates ( *P* = 0.781). Acute post-TPS bleeding did not differ significantly among the incision groups (7.7% vs. 9.4% vs. 10.0%; *P* = 0.940).

**Table:** **Table 2** Clinical and technical factors associated with successful bile duct cannulation per TPS session.

**Parameter**	**Session number**	**Successful cannulation**	**Successful rate**
**TPS session**	115	86	74.8%
**Sex**			
Male	53 (46.1%)	44 (48.8%)	83.0%
Female	62 (53.9%)	42 (51.2%)	67.7%
**Age**			
> 50	96 (83.5%)	71 (82.6%)	74.0%
≤ 50	19 (16.5%)	15 (17.4%)	78.9%
**Indication**			
Biliary stone	90 (78.3%)	64 (74.4%)	71.1%
Distal obstruction	13 (11.3%)	12 (14.0%)	92.3%
Proximal obstruction	10 (8.7%)	9 (10.5%)	90.0%
Others	2 (1.7%)	1 (1.2%)	50.0%
Macroscopic appearance of the ampulla Vater*			
Type 1	34 (29.6%)	27 (31.4%)	79.4%
Type 2	19 (16.5%)	9 (10.5%)	47.4%
Type 3	32 (27.8%)	27 (31.4%)	84.4%
Type 4	30 (26.1%)	23 (26.7%)	76.7%
**Periampullary diverticulum†**			
No diverticulum	77 (67.0%)	57 (66.3%)	74.0%
Type I, IIa, IIb	22 (19.1%)	19 (22.1%)	86.4%
Type III	16 (13.9%)	10 (11.6%)	62.5%
**Endoscopist**			
High volume	57 (49.6%)	44 (51.2%)	77.2%
Low volume	58 (50.4%)	42 (48.8%)	72.4%
**TPS incision exposure**			
Inadequately exposed	53 (46.1%)	35 (40.7%)	66.0%
Well-exposed	62 (53.9%)	51 (59.3%)	82.3%
**TPS incision extent**			
Short incision	52 (45.2%)	31 (36.0%)	59.6%
Long incision	53 (46.1%)	46 (53.5%)	86.8%
Full incision	10 (8.7%)	9 (10.5%)	90.0%
**Acute post-TPS bleeding**			
No	105 (91.3%)	77 (89.5%)	73.3%
Yes	10 (8.7%)	9 (10.5%)	90.0%
*Type 1: regular papilla; Type 2: small papilla, often flat, with a diameter ≤ 3 mm; Type 3: protruding or pendulous papilla; Type 4: creased or ridged papilla. †Papilla located completely inside the diverticulum (type I), papilla located in the inner (type IIa) and outer (type IIb) margins of the diverticulum, and papilla located outside the diverticulum (type III). TPS, transpancreatic precut sphincterotomy.			

At the session level, Table 3 summarizes associations between patient or technical factors and biliary cannulation outcomes. In univariate analysis, older age, male sex, a high-volume endoscopist, ERCP indication, periampullary diverticulum, and acute bleeding were not significantly associated with success. Type 2 papillae (odds ratio [OR] 0.200, *P* = 0.011), well-exposed incisions (OR 2.384, *P* = 0.049), and long incisions (OR 4.452, P = 0.003) were significantly associated with cannulation success. In multivariate analysis, type 2 papillae (OR 0.189, *P* = 0.003) and long incisions (OR 3.711, *P* = 0.010) remained independently associated with success. These findings were unchanged in sensitivity analyses adjusting for examiner volume and calendar time (Supplementary Table 1 and Supplementary Table 2).

**Table:** **Table 3** Univariate and multivariate analyses of factors associated with successful biliary cannulation per TPS session.

**Variable**		**Univariate analysis**		**Multivariate analysis**	
		**OR (95% CI)**	***P* value **	**OR (95% CI)**	***P* value **
Age	< 50 years	Reference			
	> 50 years	0.757 (0.230–2.498)	0.648		
Sex	Female	Reference			
	Male	2.328 (0.953–5.687)	0.064		
Endoscopist	Low volume	Reference			
	High volume	1.289 (0.554–3.002)	0.556		
Indication	Biliary stone	Reference			
	Distal stricture	4.875 (0.603–39.426)	0.137		
	Proximal stricture	3.656 (0.441–30.329)	0.230		
	Others	0.406 (0.024–6.741)	0.530		
Macroscopic appearance of the ampulla Vater*	Type 1	Reference		Reference	
	Type 2	0.200 (0.058–0.695)	0.011	0.189 (0.062–0.575)	0.003
	Type 3	0.685 (0.367–4.609)	0.685		
	Type 4	0.798 (0.263–2.792)	0.798		
Periampullary diverticulum†	No diverticulum	Reference			
	Type I, IIa, IIb	2.222 (0.594–8.318)	0.236		
	Type III	0.585 (0.188–1.816)	0.353		
TPS incision exposure	Inadequately exposed	Reference			
	Well-exposed	2.384 (1.004–5.661)	0.049		
TPS incision extent	Short incision	Reference		Reference	
	Long incision	4.452 (1.689–11.732)	0.003	3.711 (1.370–10.050)	0.010
	Full incision	6.097 (0.718–51.765)	0.098	6.097 (0.718–51.765)	
Acute post-TPS bleeding	No bleeding	Reference		Reference	
	Bleeding	3.273 (0.396–27.015)	0.271	3.273 (0.396–27.015)	
*Type 1: regular papilla; Type 2: small papilla, often flat, with a diameter ≤ 3 mm; Type 3: protruding or pendulous papilla; Type 4: creased or ridged papilla. †Papilla located completely inside the diverticulum (type I), papilla located in the inner (type IIa) and outer (type IIb) margins of the diverticulum; and papilla located outside the diverticulum (type III). CI, confidence interval; OR, odds ratio; TPS, transpancreatic precut sphincterotomy.					

At the ERCP level, Table 4 summarizes outcomes after the initial TPS incision. Because long and full incisions provide a similar degree of exposure, they were analyzed together as a single long/full group. Overall biliary cannulation rates were comparable between short and long/full incisions ( *P* = 0.825). After the first incision, the long/full group had a higher cannulation rate (92.9% vs. 56.0%; *P* < 0.001) and fewer additional incisions (4.8% vs. 42.0%; *P* < 0.001). Time to CBD cannulation was shorter for long/full incisions (25.1 ± 9.3 vs. 31.4 ± 16.9 minutes; *P* = 0.026) and total ERCP duration was similar between groups (46.7 ± 15.8 vs. 51.1 ± 21.8 minutes; *P* = 0.288). ERCP-related complications did not differ significantly between groups. PEP occurred in seven of 92 ERCP procedures (7.6%). Exploratory ERCP-level univariable analysis did not reveal any statistically significant associations between clinical or procedural variables and PEP (Supplementary Table 3).

**Table:** **Table 4** ERCP outcomes per procedure according to the extent of the initial TPS incision.

**Outcome**	**Short incision (n = 50)**	**Long/full incision (n = 42)**	***P* value **
Successful cannulation	47 (94.0%)	39 (92.9%)	1.000 *
Successful cannulation after first incision	28 (56.0%)	39 (92.9%)	< 0.001*
Additional incision	21 (42.0%)	2 (4.8%)	<0.001*
Cannulation time (min)	31.4 (16.9)	25.1 (9.3)	0.026†
ERCP time (min)	51.1 (21.8)	46.7 (15.8)	0.288†
Post-ERCP pancreatitis	2 (4.0%)	5 (11.9%)	0.240*
Acute post-TPS bleeding	4 (8.0%)	3 (7.1%)	1.000*
Requiring hemostasis	3 (6.0%)	5 (11.9%)	0.462*
Delayed bleeding	2 (4.0%)	3 (7.1%)	0.657*
Cholangitis	1 (2.0%)	3 (7.1%)	0.328*
*P value derived from Fisher’s exact test. †P value derived from Student’s t test; Values are presented as the number (%) or mean ± standard deviation. ERCP, endoscopic retrograde cholangiopancreatography; TPS, transpancreatic precut sphincterotomy.			

Given the limited number of PEP events, multivariable analysis was not performed.

Endoscopic images clearly demonstrated both biliary and pancreatic duct orifices in 82 patients; the remaining 10 cases were excluded because post-TPS orifice positions were not evaluable. Among the evaluable cases, type B was the most common pattern (65.9%), with the bile duct orifice located in the upper-left region of the pancreatic orifice within twice the sphincterotome width. Type C accounted for 19.5% and type A for 14.6% ( Table 5 ).

**Table:** **Table 5** Distribution of the relative positions of the biliary and pancreatic duct orifices.

**Total measurable cases**	**Type A**	**Type B**	**Type C**
82	12 (14.6%)	54 (65.9%)	16 (19.5%)
Type A: Upper left with a distance exceeding twice the width of the sphincterotome. Type B: Upper left with a distance less than twice the width of the sphincterotome. Type C: Left and lower left.			

## Discussion

In this retrospective study, TPS provided reliable biliary access once the pancreatic duct had been cannulated. Biliary cannulation outcomes were comparable across ERCP indications, periampullary diverticulum status, and most papillary morphologies. At the session level, Haraldsson type 2 papillae were associated with lower success, whereas longer TPS incisions improved cannulation rates. Procedures beginning with a long/full incision also demonstrated shorter cannulation times. After TPS, the bile duct orifice was typically located in the upper-left region relative to the pancreatic orifice.

### Patient factors associated with successful cannulation by TPS

Prior studies have investigated how patient characteristics influence selective biliary cannulation, including indication 11 , papilla morphology 12 , periampullary diverticula 12 13 , and altered anatomy 14 . Although a meta-analysis based on the Haraldsson classification reported no major differences in cannulation failure across papillary types 15 , papilla morphology has been linked to variable outcomes in needle-knife precut sphincterotomy, with type 3 papillae showing higher success and type 2 lower success 16 . In our study, type 2 papillae were associated with reduced TPS success, whereas other patient characteristics and acute post-TPS bleeding were not significantly associated with successful cannulation. A previous TPS series in “small papillae” reported a high success rate (98.2%) 17 , although “small papillae” referred to a small papillary oral protrusion rather than the ≤ 3-mm definition used in the Haraldsson system. Type 2 papillae remain relatively uncommon, comprising 8.7% to 14% of cases in prior studies and in our cohort 8 11 . Previous reports have also suggested that needle-knife precut may be affected by periampullary diverticula 15 18 or acute active bleeding during precut 19 . In contrast, these factors were not associated with TPS outcomes in our analysis once pancreatic duct access was obtained. Reduced TPS success in Haraldsson type 2 papillae has not been previously reported. A recent study did not demonstrate a significant association between papilla morphology and TPS success 20 . The underlying mechanism remains unclear. The small orifice and relatively flat configuration of type 2 papillae may be associated with reduced septal prominence, making the bile duct orifice more difficult to identify after the incision. Further studies are needed to clarify the anatomical basis of this association.

### Technical factors associated with successful cannulation by TPS

Goff originally recommended a short incision of no more than 5 mm, with stepwise extension only if needed, reporting a 94% overall cannulation rate without bleeding complications 5 . Subsequent studies have described differing techniques and outcomes. Akashi et al. reported moderate to large incisions extending beyond the first transverse fold, with an initial success rate of 60% and a 1.2% delayed bleeding rate 21 . In contrast, Halttunen et al. described full incisions similar to standard biliary sphincterotomies, achieving a 97.3% success rate and a 1.6% delayed bleeding rate 22 . However, these reports focused on individual techniques, and direct comparisons of different incision strategies remain limited. In our cohort, where no fixed protocol for incision length was applied, longer TPS incisions, defined as those reaching at least the midpoint of the papillary oral protrusion, were associated with higher biliary cannulation rates than short incisions were, without an increase in bleeding among long/full incisions. Visual classification of incision extent showed substantial interobserver agreement, supporting consistency of these findings.

### Clinical impact of incision extent

At the ERCP level, procedures beginning with a long/full TPS incision achieved higher first-incision cannulation rates and shorter cannulation times, although overall success was similar across incision groups. Although Goff recommended starting with a short incision to reduce complications, AE rates in our cohort did not differ between short and long/full incisions. Advanced cannulation techniques often require more time and procedure steps, which may lengthen duration of ERCP and increase the burden of anesthesia 23 . In our cohort, initiating TPS with a longer incision reduced the need for additional extensions and, therefore, improved procedure efficiency.

Safety should also be considered. Although our TPS data did not show a clear association between pancreatic-side incision length and bleeding, a prior study of biliary sphincterotomy reported higher bleeding rates with more extensive cuts 24 . In our classification, a “long” incision extends beyond the midpoint of the papillary oral protrusion but remains substantially shorter than a “full” incision reaching the superior margin. Endoscopically, the superior margin of the papillary protrusion may not always be clearly delineated, which can make a full incision technically more difficult to control and potentially increase risk of overextension. In contrast, the midpoint is generally more readily identifiable, providing a practical and reproducible landmark during the TPS. In our cohort, this degree of extension was not associated with increased bleeding or perforation, suggesting that extension beyond the midpoint may represent a practical balance among procedure effectiveness, safety, and technical control. Because long and full TPS incisions have similar success rates, beginning with a long incision may be sufficient for most cases, with full extension reserved for selected situations. In our cohort, TPS was performed by both high- and low-volume endoscopists, and cannulation success was not significantly associated with operator volume, suggesting that, within the context of experienced ERCP operators at our center, this strategy may not be restricted to exclusively high-volume endoscopists. Although the reported bleeding risks associated with short and full incisions were low (1.2% 21 and 1.6% 22 , respectively) in two separate studies, an initial short incision followed by cautious incremental extension may still be considered during the early learning phase of the TPS in clinically stable patients. Further studies are needed to define the optimal incision extent.

### Locating the bile duct after TPS

Following TPS, identifying the bile duct orifice can present an additional technical challenge. Among the six unsuccessful cases, all had a well-exposed incision at the end of ERCP. Two patients later underwent repeat ERCP, in which the bile duct orifice was found centrally within the prior incision without need for further cutting. In our cohort, most orifices (65.9%) were located in the upper-left quadrant and within two sphincterotome widths of the pancreatic orifice, which offer a useful reference point when the orifice is not immediately visible. A smaller proportion (14.6%) were located farther away, suggesting that only a minority of patients may require a more extensive incision. Theoretically, post-TPS position of the bile duct orifice may vary according to the length of the pancreatobiliary junction 25 and may also be influenced by the extent of the pancreatic sphincterotomy. In the exploratory evaluation, no clear pattern of association was observed between protrusion length and post-TPS orifice configuration. Taken together, these findings suggest that only a minority of patients may require a more extensive incision. In such cases, careful stepwise extension is advised to minimize risk of overextension and potential perforation.

### Practical implications

Although TPS may appear straightforward, determining appropriate incision extent and locating the bile duct orifice can be challenging, particularly for low-volume endoscopists and beginners. In clinical practice, TPS may be considered in patients with difficult bile duct cannulation and unintended pancreatic duct access, especially when performed by endoscopists familiar with core ERCP techniques and competent in biliary sphincterotomy. On the basis of our findings, a long initial incision extending beyond the midpoint of the papillary protrusion may represent a reasonable balance between procedure effectiveness and technical control. When the bile duct orifice is not immediately visible after TPS, initial probing can start in the upper-left direction relative to the pancreatic orifice, typically within approximately two sphincterotome widths. Full incision may be reserved for selected cases in which cannulation remains unsuccessful despite a long incision. During the early learning phase, an initial short incision followed by cautious stepwise extension may be appropriate in clinically stable patients because this approach may prolong the cannulation time.

### Limitations

This study has several limitations. First, its retrospective single-center design may introduce selection bias. Second, the midpoint of the papillary oral protrusion used to categorize incision extent represents a practical endoscopic landmark rather than an anatomical reference, yet it was readily identifiable and showed substantial interobserver agreement. Third, because this was an image-based review, the initial and final TPS incisions were selected for analysis because they represented the most consistently documented steps within each procedure. Although intermediate adjustments were not included in the final analysis, the available images were sufficient to allow reliable classification of the incision extent and exposure. In addition, because incision extent was assessed using post-incision endoscopic images, complete blinding to procedure outcomes was not fully feasible, which may introduce potential assessment bias. Fourth, because TPS was introduced at our institution in 2016, a potential learning curve effect cannot be entirely excluded. Although calendar time was not independently associated with TPS session success in the sensitivity analysis, residual temporal effects may still be possible. In addition, because incision extent was determined by the operating endoscopist on the basis of real-time procedure judgment, it was not randomly assigned and may reflect operator-related factors that influence TPS success. Although multivariable adjustment and sensitivity analysis accounting for examiner volume were performed, residual confounding cannot be entirely excluded. Despite these limitations, these observations may help guide future prospective studies evaluating whether specific endoscopic landmarks can contribute to procedure standardization or training in the TPS.

## Conclusions

TPS success is determined primarily by technical factors, with type 2 papillae being the main patient-related challenge. Longer incisions, defined as those reaching the midpoint of the papillary oral protrusion or higher, improved cannulation success. After the TPS, the bile duct orifice most commonly appeared in the upper-left region of the pancreatic orifice.

## Supplementary Material

Supplementary Material Zusatzmaterial
