# Supplementary material for: Technical factors associated with biliary cannulation success after transpancreatic precut sphincterotomy
Source: Endosc Int Open. 2026 May 19;14:a28631217. doi: 10.1055/a-2863-1217 (PMC13289974; doi:10.1055/a-2863-1217)
Supplement: Supplementary file 1 — Supplementary Material [file 10-1055-a-2863-1217_28644561.pdf]

Supplementary Table 1 Sensitivity analysis of factors associated with TPS session success with forced inclusion of endoscopist volume.

**Variable**	**OR**	**95% CI**	***P* value**
Long incision (vs. short incision)	3.696	1.363-10.020	0.010
Papilla type 2 (vs. papilla type 1)	0.191	0.062-0.583	0.004
High-volume endoscopist (vs. low-volume)	1.081	0.431-2.708	0.869

Endoscopist volume was forcibly entered into the multivariable model as a sensitivity analysis to assess potential operator-related confounding.
CI, confidence interval; OR, odds ratio; TPS, transpancreatic sphincterotomy.

Supplementary Table 2 Sensitivity analysis of factors associated with TPS session success adjusted for calendar time

**Variable**	**OR**	**95% CI**	***P* value**
Long incision (vs. short incision)	3.941	1.431–10.856	0.008
Papilla type 2 (vs. papilla type 1)	0.175	0.057–0.541	0.002
Calendar time	1.140	0.914–1.421	0.245

Calendar time was entered as an ordinal variable (coded 1–7) representing sequential annual intervals from TPS introduction.
CI, confidence interval; OR, odds ratio; TPS, transpancreatic sphincterotomy.

Endosc Int Open | DOI 10.1055/a-2863-1217 | © The Author(s).

Supplementary Table 3 Factors associated with post-ERCP pancreatitis in TPS cases (ERCP level, n=92).

**Variable**	**No post-ERCP pancreatitis (n = 85)**	**Post-ERCP pancreatitis (n = 7)**	***P* value**
**Sex**			
Male	43 (50.6%)	4 (57.1%)	1.000§
**Age**			
> 50	71 (83.5%)	4 (57.1%)	0.115§
**Indication**			
Biliary stone	62 (72.9%)	7 (100.0%)	0.471¶
Distal obstruction	12 (14.1%)	0 (0.0%)	
Proximal obstruction	9 (10.6%)	0 (0.0%)	
Others	2 (2.4%)	0 (0.0%)	
**History of biliary pancreatitis**	13 (15.3%)	1 (14.3%)	1.000§
**End-stage renal disease**	2 (2.4%)	1 (14.3%)	0.213§
**Papilla morphology***			0.665¶
Type 1	25 (29.4%)	3 (42.9%)	
Type 2	14 (16.5%)	0 (0.0%)	
Type 3	25 (29.4%)	2 (28.6%)	
Type 4	21 (24.7%)	2 (28.6%)	
**Periampullary diverticulum†**			0.607¶
No diverticulum	56 (65.9%)	6 (85.7%)	
Type I	1 (1.2%)	0 (0.0%)	
Type IIa	6 (7.1%)	1 (14.3%)	
Type IIb	12 (14.1%)	0 (0.0%)	
Type III	10 (11.8%)	0 (0.0%)	
**Endoscopist**			1.000§
Low volume	43 (50.6%)	3 (42.9%)	
High volume	42 (49.4%)	4 (57.1%)	
**Successful cannulation**	79 (92.9%)	7 (100.0%)	1.000§
**Initial TPS success**	61 (71.8%)	6 (85.7%)	0.669§
**Pancreatic stent placed**	77 (90.6%)	7 (100.0%)	1.000§
**Peri-ERCP hydration**	56 (65.9%)	3 (42.9%)	0.245§
**Rectal NSAIDs**	36 (42.4%)	2 (28.6%)	0.695§
**Initial TPS‡ incision extent**			0.351¶
Short incision	48 (56.5%)	2 (28.6%)	
Long incision	31 (36.5%)	4 (57.1%)	
Full incision	6 (7.1%)	1 (14.3%)	
**Final TPS‡ incision extent**			0.560¶
Short incision	29 (34.1%)	1 (14.3%)	
Long incision	47 (55.3%)	5 (71.4%)	
Full incision	9 (10.6%)	1 (14.3%)	
**Initial incision exposure**			
Inadequate	44 (51.8%)	3 (42.9%)	0.711§
Well-exposed	41 (48.2%)	4 (57.1%)	
**Final incision exposure**			
Inadequate	33 (38.8%)	2 (28.6%)	0.705§

Well-exposed	52 (61.2%)	5 (71.4%)

*Type 1: regular papilla; Type 2: small papilla, often flat, with a diameter ≤ 3 mm; Type 3: protruding or pendulous papilla; Type 4: creased or ridged papilla.
†Papilla located completely inside the diverticulum (type I), papilla located in the inner (type IIa) and outer (type IIb) margins of the diverticulum, and papilla located outside the diverticulum (type III).
‡Transpancreatic precut sphincterotomy.
§Derived from Fisher’s exact test.
¶Derived from the chi-square test
CI, confidence interval; ERCP, endoscopic retrograde cholangiopancreatography; OR, odds ratio; PEP, post-ERCP pancreatitis; TPS, transpancreatic precut sphincterotomy.
